# Pancreatic regional blood flow links the endocrine and exocrine diseases

**DOI:** 10.1172/JCI166185

**Published:** 2023-08-01

**Authors:** Adam A. Rizk, Michael P. Dybala, Khalil C. Rodriguez, Marjan Slak Rupnik, Manami Hara

**Affiliations:** 1 Department of Medicine, The University of Chicago, Chicago, Illinois, USA.; 2 Center for Physiology and Pharmacology, Medical University of Vienna, Vienna, Austria.

**Keywords:** Endocrinology, Islet cells

## Abstract

An increasing number of studies have demonstrated that disease states of the endocrine or exocrine pancreas aggravate one another, which implies bidirectional blood flow between islets and exocrine cells. However, this is inconsistent with the current model of unidirectional blood flow, which is strictly from islets to exocrine tissues. This conventional model was first proposed in 1932, and it has never to our knowledge been revisited to date. Here, large-scale image capture was used to examine the spatial relationship between islets and blood vessels in the following species: human, monkey, pig, rabbit, ferret, and mouse. While some arterioles passed by or traveled through islets, the majority of islets had no association with them. Islets with direct contact with the arteriole were significantly larger in size and fewer in number than those without contact. Unique to the pancreas, capillaries directly branched out from the arterioles and have been labeled as “small arterioles” in past studies. Overall, the arterioles emerged to feed the pancreas regionally, not specifically targeting individual islets. Vascularizing the pancreas in this way may allow an entire downstream region of islets and acinar cells to be simultaneously exposed to changes in the blood levels of glucose, hormones, and other circulating factors.

## Introduction

The pancreas is a unique organ composed of exocrine and endocrine tissues, in which the former produces enzymes for digestion and the latter secretes metabolic hormones. The majority of the pancreas consists of exocrine tissues, and endocrine cells forming the islets comprise only 1%–2% of the organ. Since the first description of the islet blood supply in 1932 (1), the pancreatic islet has been considered an enclosed micro-organ that receives a dedicated arteriole (2–6). According to this model, blood flows through the islet completely separated from the exocrine component or forms an insulo-acinar portal system before it drains from 1 or more venules. In fact, exocrine and endocrine compartments of the pancreas have been studied by different scientific communities, and the diseases of the exocrine and endocrine pancreas are treated by physicians in different medical disciplines: gastroenterologists and endocrinologists, respectively. Notably, it is still not clear why these 2 systems combine into 1 organ.

In pancreas-associated transcription factor 1a–deficient (Ptf1a-deficient) mice lacking the exocrine pancreas, endocrine cells home into the spleen (7). Interestingly, these cells were singly scattered throughout the spleen, suggesting that the exocrine tissues are not required for endocrine cell survival but are necessary for islet formation. In Pdx1-*Kras*–expressing *ob/ob* mice, obesity led to aberrant cholecystokinin (CCK) expression in islet cells, which accelerated *Kras*-driven pancreatic ductal tumorigenesis (8). Similarly in obese and diabetic *db/db* mice, islet-derived CCK induced upregulation of trypsin family genes and mTOR activity in peri-islet acinar cells, which in turn may facilitate islet expansion (9).

Clinically, it has been long known that patients with type 1 diabetes (T1D) have smaller pancreata and reduced exocrine function compared with nondiabetic controls (10–15), which implies that insulin or some other β cell component may serve as a growth or survival factor for the exocrine tissues (16, 17). Intriguingly, a very recent study demonstrated that individuals with insulin gene mutation, causing severe insulin deficiency without autoimmunity, have a smaller pancreas (18). This genetics case suggests that insulin is trophic for acinar cells and that insulin deficiency directly causes a reduction in exocrine pancreas size. It has an important implication for T1D, as the authors stated, that a local effect could lead to diminished exocrine pancreas size before obvious systemic insulin insufficiency leading to elevated glucose levels. Type 3c diabetes (T3cD) refers to diabetes following pancreatic diseases such as pancreatitis, pancreas removal, cystic fibrosis (CF), and hemochromatosis (19). Patients with T3cD need treatment for digestive problems, including replacement of pancreatic enzymes and the fat-soluble vitamins A, D, E, and K. Causal mechanisms of CF-related diabetes (CFRD) have long been thought to be β cell intrinsic, since it was granted that CF transmembrane conductance regulator (CFTR) was expressed in β cells (20). However, the CFTR protein is highly expressed in the pancreatic duct epithelia (21, 22), and deficiencies of ductal fluid secretion begin in utero in patients with CF (23). In fact, immune-reactive trypsinogen in serum is used as a marker for the neonatal screening test for CF during an asymptomatic period, suggesting pancreatic involvement in the early life of patients with CF. It is well known that CFRD has an unusual phenotype accompanied by insulin resistance, particularly during acute pulmonary exacerbations (24). Impairment of duct cell functions may trigger pancreatic inflammation (17).

The field has started to recognize the close interaction between the exocrine and endocrine pancreas from studies of animal models as well as humans. Exocrine blood supply has only been described through gross characteristics of the whole pancreas, which does not include the islet blood supply (25). Our recent model of bidirectional blood flow between the islet and exocrine tissues physically links both compartments (26). In this study, we specifically examined the spatial relationship between the pancreatic arteriole and nearby islets in 3D in 6 different species. Here, we report that arterioles formed tree-like branches and fed the pancreas regionally, with no evidence of separate blood supplies for the endocrine and exocrine pancreas. Islets were in the vicinity, but the majority had no contact with the arteriole. Capillaries directly branched out from arterioles, which could feed the islet from the periphery and inside of it. This new model implies rapid regional diffusion of blood flow to both compartments at the same time.

## Results

### 3D reconstruction of the microenvironment surrounding islets).

**Figure 1:**
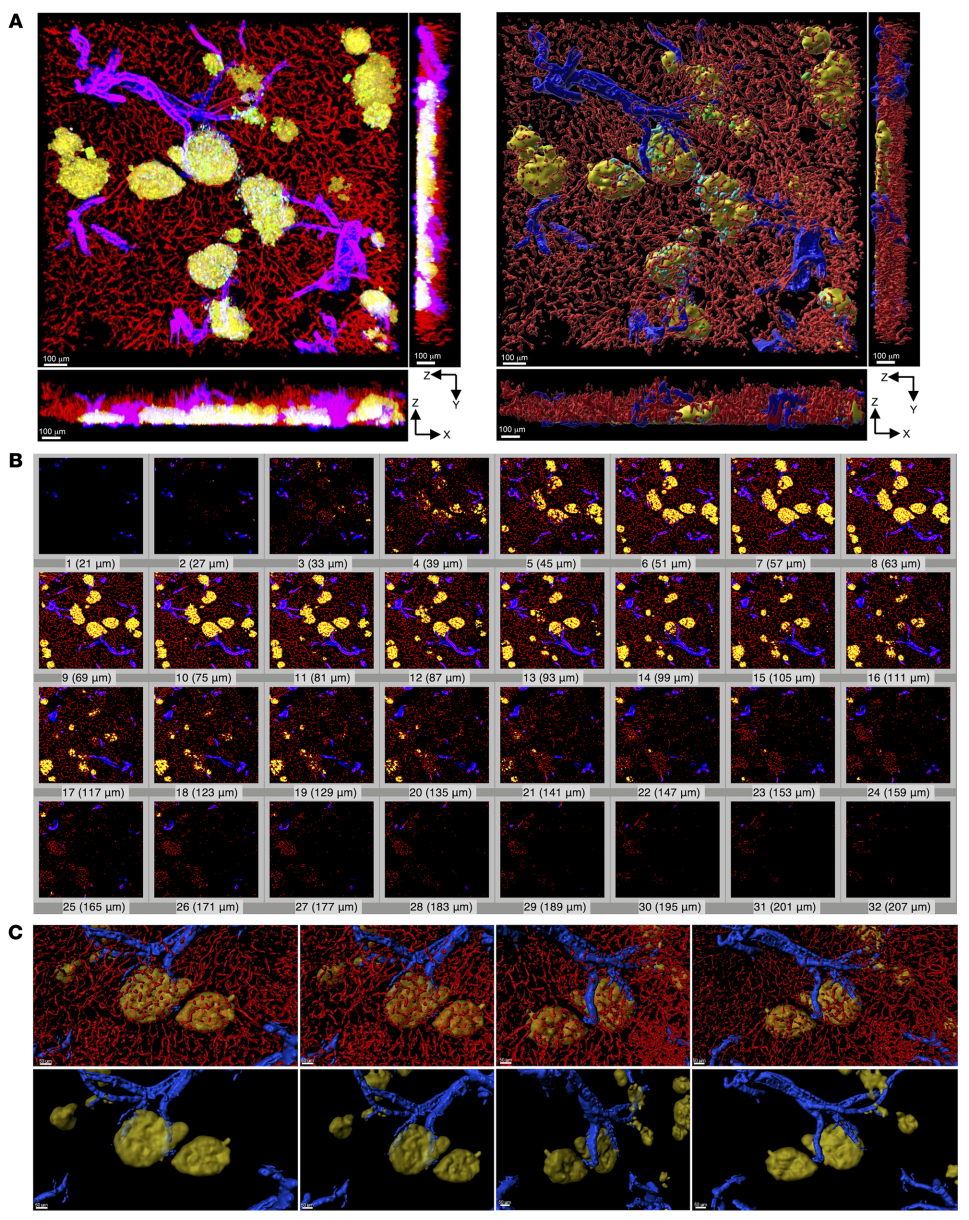
3D reconstruction of the microenvironment surrounding islets. (**A**) In situ 3D views of islets, arterioles, and surrounding exocrine tissues in X-Y, X-Z, and Y-Z planes. Left: Fluorescence images. Insulin (green), glucagon (yellow), somatostatin (cyan), CD31 (red) and α-SMA (blue). Note that in the Z axis (X-Z and Y-Z), overlapping fluorescent signals in the 3D image appear as mixed colors. Right: 3D surface-rendered images in the X-Y, X-Z, and Y-Z planes. Scale bars: 100 μm. (**B**) A stack of sequential images (images 1–32) with an increment of 6 μm used to create the images in **A** in 3D (refer to **A** for scale). (**C**) Closer views of arteriole branches and nearby islets from different angles in 3D. Top: Islets and arterioles only. Bottom: Islets, arterioles, and capillaries. Scale bars: 50 μm.

It is important that network structures such as the vasculature be analyzed in 3D, because in 2D, they merely show up as dots and short lines. Furthermore, even for 3D imaging (by a stack of serial 2D confocal images), an X-Y plane only depicts a maximum projection view from 1 angle. In 1932, Wharton (1) modeled islet blood flow as the arteriole tree supplying all individual islets in each pancreatic lobe, which is a commonly held view to this day. The author used thick pancreatic tissues and advanced techniques at the time, such as cardiac perfusion and tissue clearing by Spalteholz’s method. However, the spatial relationship between islets and vasculature was interpreted in 2D, which does not differ from current methods of image analysis in the field. In this study, fluorescence images were surface rendered in 3D, an approach suitable for tracing structures with complex branching and topology such as the vascular network. An orthogonal projection of a fluorescence image in Figure 1A (left) shows an X-Y plane along with X-Z and Y-Z planes, which is 3D surface rendered in Figure 1A (right). A stack of serial optical panels with an increment of 6 μm was used to reconstruct the captured structures into a 3D image (Figure 1B).

With this approach, we revisited the islet microcirculation, specifically focusing on the spatial interconnection between islets and arterioles. Arterioles were identified according to a well-referred book chapter written by Silverthorn (27) that was adapted from Burton (28). Vasculature can be immunostained using endothelial cell markers (e.g., CD31, CD34, and lectins). Vascular smooth muscle cells comprise the blood vessel wall in arteries, veins, and arterioles, but not in capillaries or venules. As a marker for smooth muscle cells, α–smooth muscle cell actin (α-SMA) has been widely used (29). Therefore, we observed costaining of antibodies for these 2 cell-type markers in arteries, veins, and arterioles. Then, arterioles could be differentiated from arteries and veins by their diameter, whose mean values differ considerably: 30 μm, 4 mm, and 5 mm, respectively. In Figure 1C, 3D surface–rendered islets and arterioles are shown from 4 different angles with and without capillaries (Figure 1C, top and bottom, respectively). They demonstrate that an observation only from a single angle can contribute to a bias (also see Supplemental Videos 1 and 2; supplemental material available online with this article; https://doi.org/10.1172/JCI166185DS1).

### In situ 3D views of islets, arterioles, and surrounding exocrine tissues in various species.

**Figure 2:**
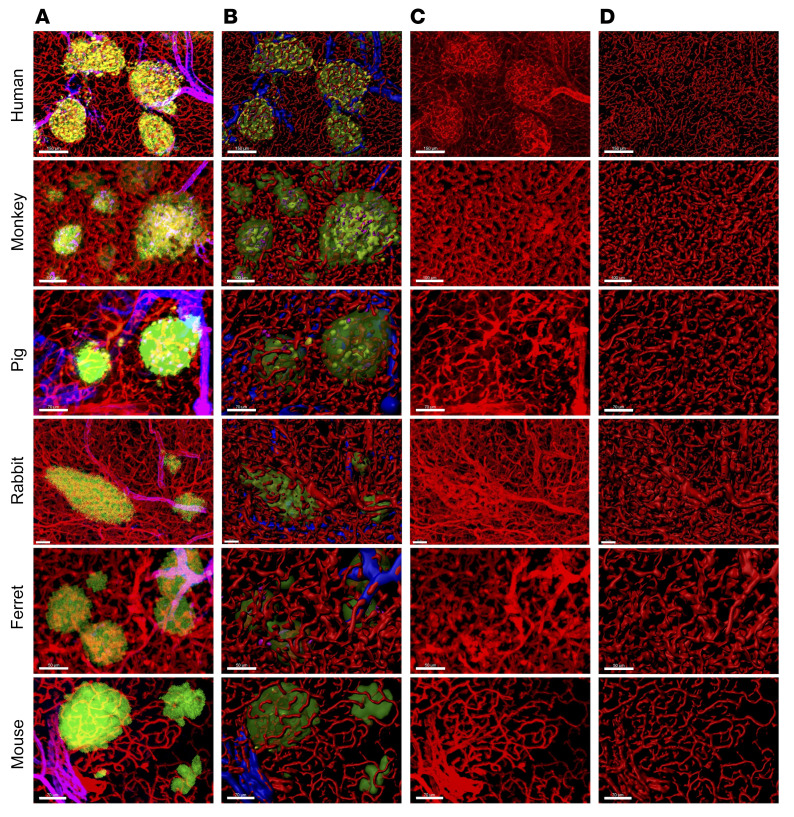
In situ 3D view of the islet microenvironment in various species. Rows from the top: human, monkey, pig, rabbit, ferret and mouse. Islets (green; HPi1 or insulin), vasculature (red; CD31 or tomato lectin), and arterioles (blue; α-SMA). (**A**) Fluorescence images. (**B**) 3D surface-rendered images. (**C**) Fluorescence images showing vasculature only. (**D**) 3D surface–rendered images showing vasculature only. Scale bars: Scale bars: human, 150 μm; monkey, 100 μm; pig, 70 μm; rabbit, 50 μm; ferret, 50 μm; mouse, 70 μm.

We have previously demonstrated bidirectional blood flow between the endocrine and exocrine pancreas in mouse pancreas by intravital recordings and analysis of individual RBC flow in 391 islets from 192 mice (26). To support the 2D analysis of these in vivo recordings, we further analyzed the structural vascular integrity of the 2 compartments in 3D in mouse and human pancreata. In the present study, in order to assess whether this integration would be generally conserved among species, we expanded our analysis to 4 additional mammalian species to cover the scaling range between mice and humans. In situ views of islets were captured in 3D from various species: human, monkey, pig, rabbit, ferret, and mouse (Figure 2; islets in green, vasculature in red, and arterioles in blue). The original fluorescence images are shown in Figure 2A and are 3D surface rendered in Figure 2B. Surface rendering is critical to take advantage of 3D imaging, which is a useful tool to visualize the morphology of a structure. This reconstruction provides insight into the spatial structure as well as its interaction with the surrounding microenvironment. In Figure 2, C and D, only vasculature is displayed. Contrary to the prevailing model of the distinct islet blood supply organization, in these close views of a few islets from each species, it did not appear that every single islet received “a dedicated arteriole.” Therefore, we expanded our study to large-scale tissue analysis to explore and deduce the vascular network structure supporting the dynamic pancreas blood flow in 3D.

### Large-scale 3D image analysis of pancreata from 6 species.

**Figure 3:**
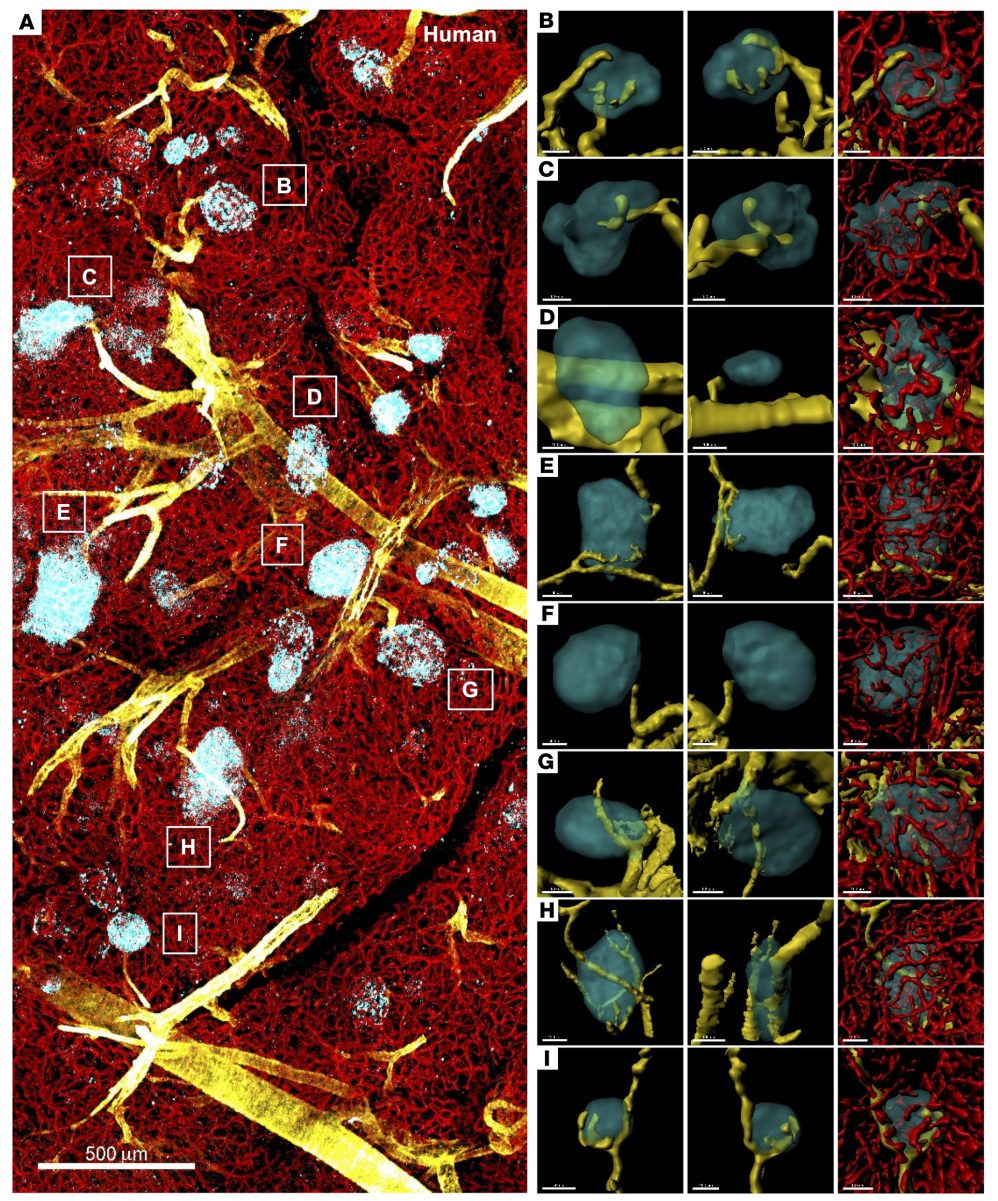
Large-scale 3D image analysis of the human pancreas. (**A**) Large-scale pancreatic tissue image showing glucagon (cyan), α-SMA (yellow), and CD31 (red). Scale bar: 500 μm. (**B**–**I**) Individual islets corresponding to the labels in **A** in different views. Islets, arterioles, and capillaries are surface rendered. Scale bars: 50 μm (**B** and **F**); 100 μm, 50 μm, 100 μm (**C**); 100 μm (**D**, **G**, and **H**); 80 μm (**E**); 50 μm, 100 μm, and 100 μm (**I**).

**Figure 4:**
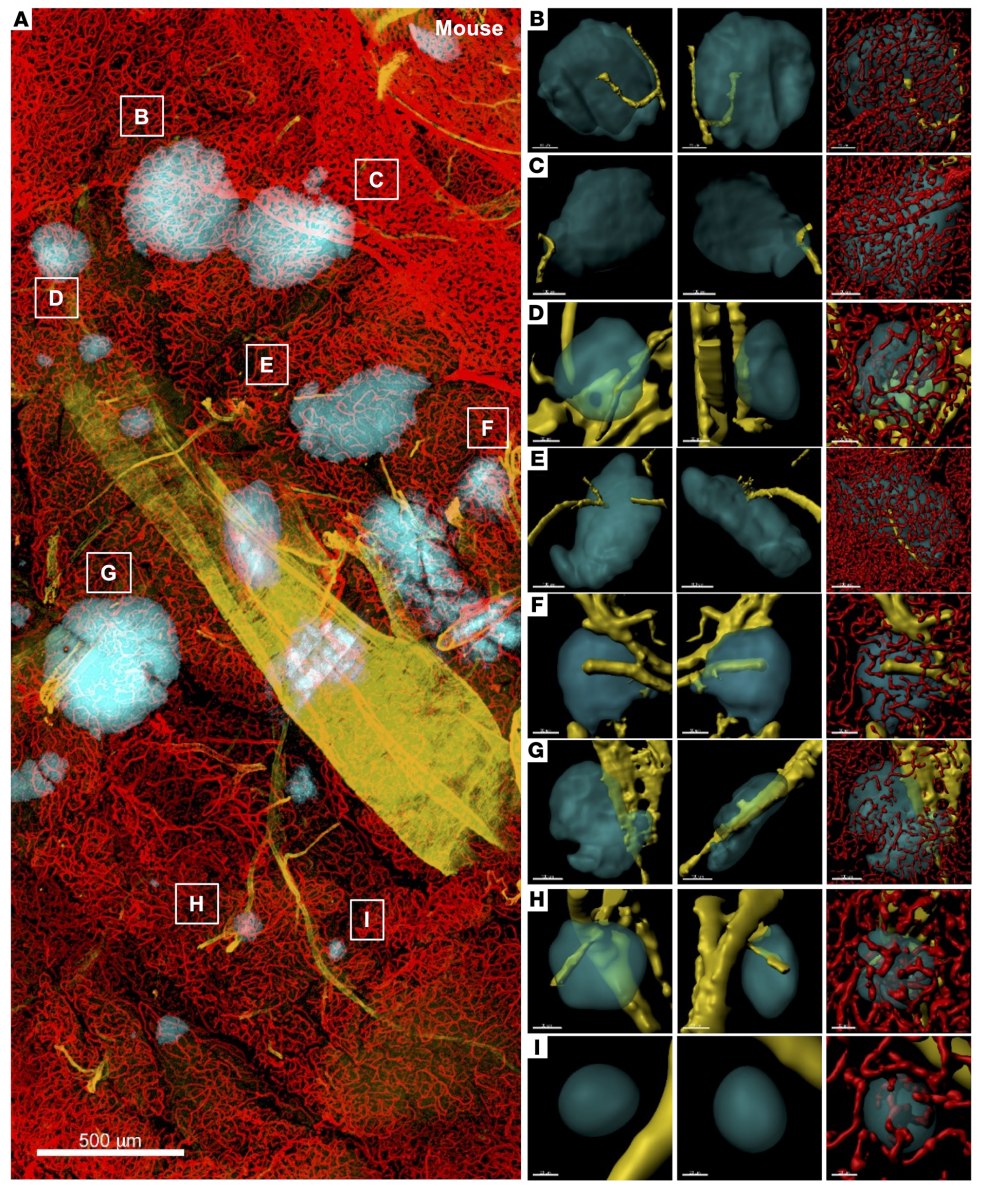
Large-scale 3D image analysis of the mouse pancreas. (**A**) Large-scale pancreatic tissue image showing insulin (cyan), α-SMA (yellow), and CD31 (red). Scale bar: 500 μm. (**B**–**I**) Individual islets corresponding to the labels in **A** in different views. Islets, arterioles, and capillaries are surface rendered. Scale bars: 80 μm, 70 μm, 70 μm (**B**); 100 μm, 100 μm, 80 μm (**C**); 50 μm (**D** and **F**); 100 μm (**E** and **G**); 30 μm (**H**); 20 μm (**I**).

The spatial relationship between α-SMA–labeled arterioles and islets was analyzed in thick pancreatic tissue slices. The image in Figure 3A shows a large area of human pancreatic tissue from a healthy 13-year-old female and captures the artery and branching arterioles. While islets were stained with insulin, glucagon, and somatostatin, α cells are highlighted in Figure 3 to show the heterogeneity in islet cell composition (such as fewer α cells in islets in B, D, and G in panel A) that we have previously reported (30). In Figure 3, B–I, staining for all 3 hormones was combined for smooth 3D surface rendering to present closer views of surface-rendered individual islets (cyan) and nearby arterioles (yellow). The first 2 columns portray the spatial orientation of islets and arterioles. It is important to examine these 2 structures together using a 360-degree manual rotation of the 3D image, since a view from 1 direction can be deceiving even in 3D images (See Supplemental Videos 1 and 2). In fact, we previously reported that as much as 85% of islets had 1 arteriole (31). This was due to counting islets from 1 angle of the 3D images while, regrettably, being biased by the prevailing concept. In the islets in Figure 3, the typical “individually dedicated arterioles” that have been described to date (1–6) were not observed. The spatial relationship of islets was in close proximity to arterioles but appeared to be rather variable. It is noteworthy that a considerable portion of arterioles appeared to supply areas of exocrine pæancreas lacking islets (Figure 3A). In the third column in Figure 3, B–I, where islets were made transparent, the integrated capillary network is exemplified. We analyzed a total of 961 islets from 5 donors (a 13-year-old female, a 16-year-old female, a 24-year-old male, a 46-year-old female, and a 57-year-old male). To determine whether the spatial arrangement of the arteriole and the islet observed in the human pancreas is conserved in other animals, we examined pancreatic tissues from 5 monkeys (*n* =772 islets), 5 pigs (*n* = 621 islets), 3 rabbits (*n* =1,109 islets), 5 ferrets (*n* =907 islets), and 6 mice (*n* =427 islets) (Supplemental Figures 1–4 and Figure 4). The configuration of these figures is the same as in Figure 3, as described above. Overall, the spatial orientation of islets and arterioles was similar throughout all 6 species, without a “dedicated” arteriole for each individual islet, and the majority of islets had no contact with the arteriole.

### Islet arteriole contact and islet size distribution.

**Figure 5:**
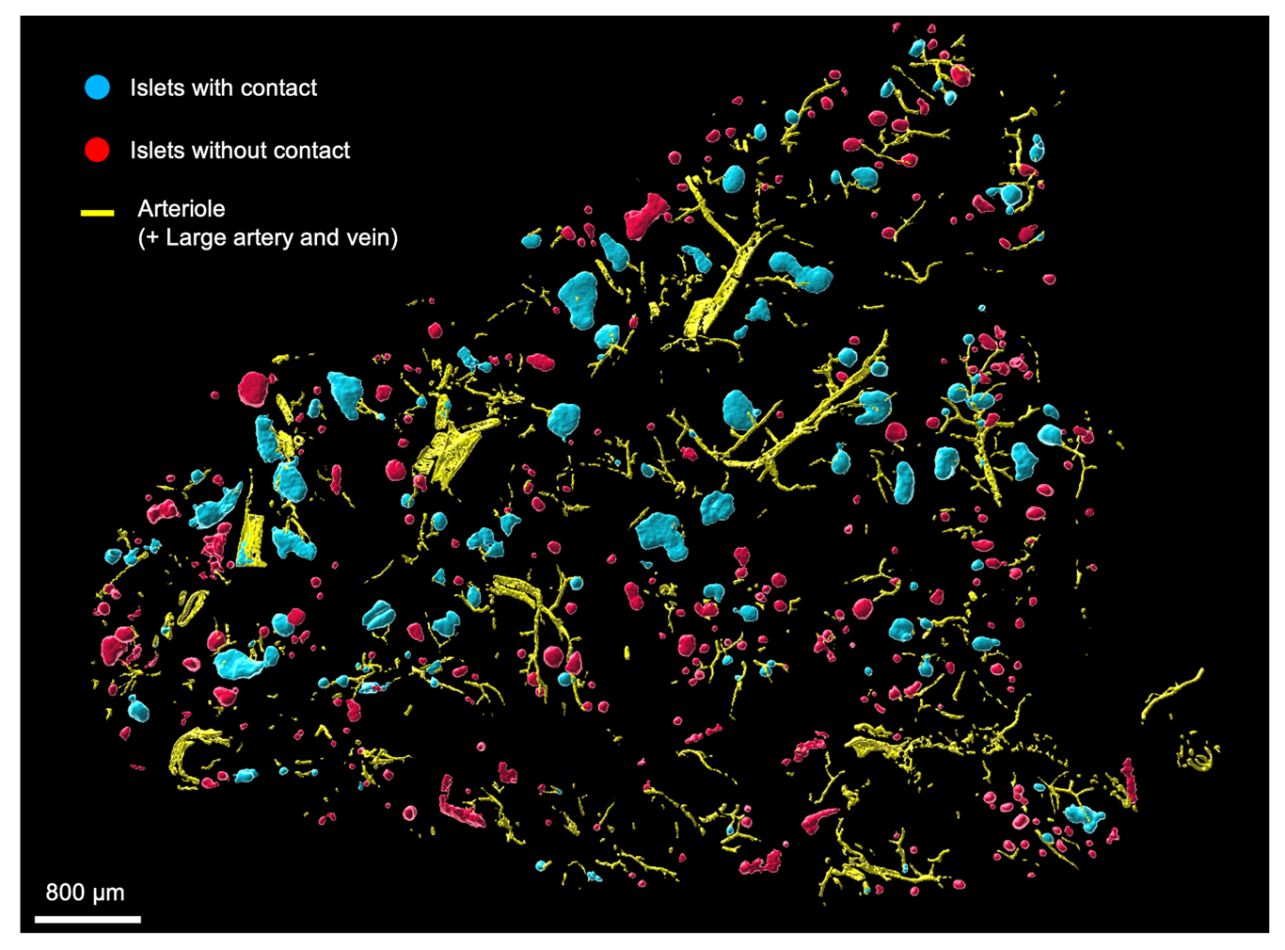
Islet arteriole contact and islet size distribution. Large 3D-rendered area of human pancreas with α-SMA–labeled arterioles (yellow), islets with arteriole contact (blue), and islets without arteriole contact (red). Scale bar: 800 μm.

**Figure 6:**
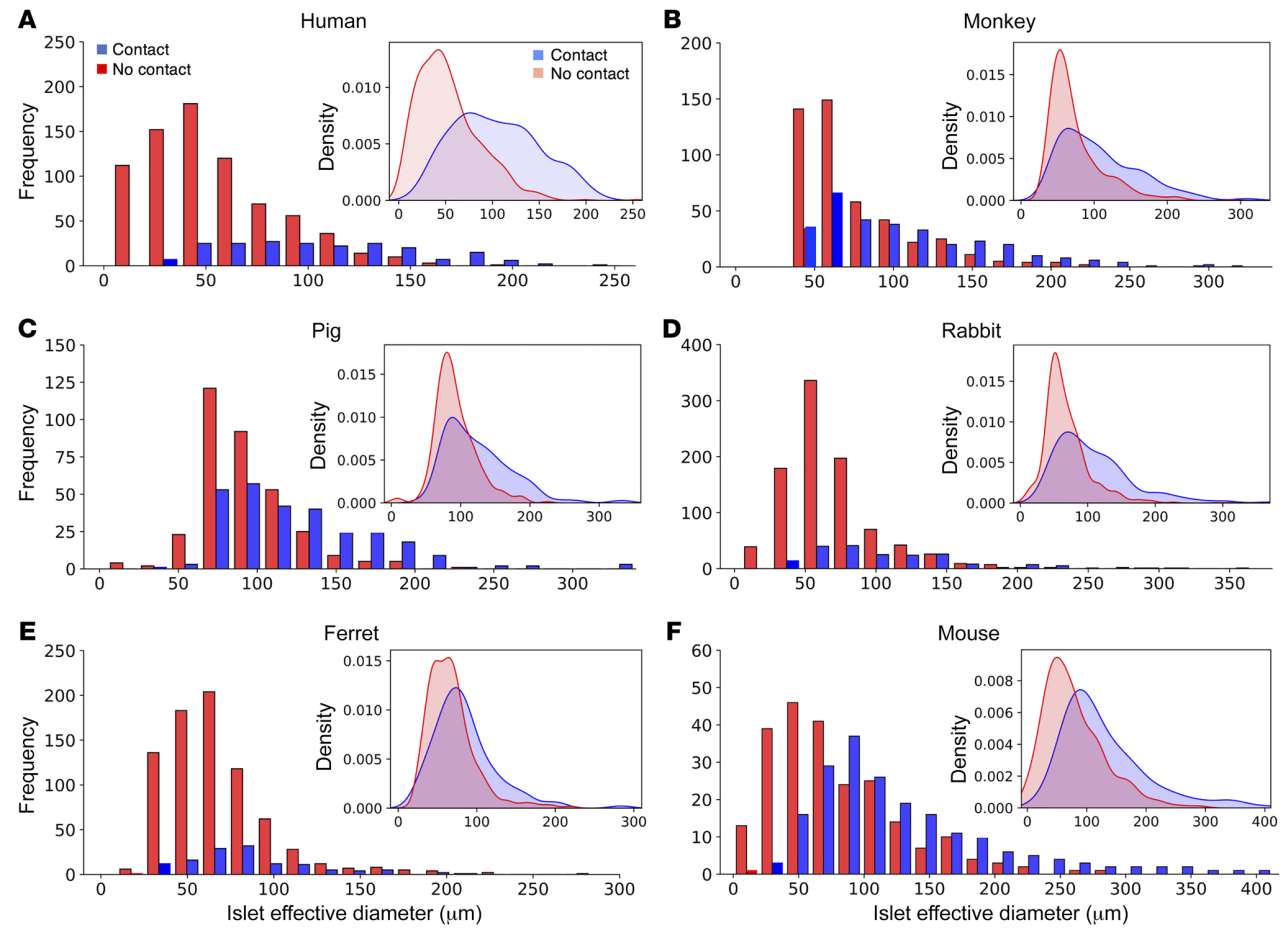
Islet size distribution and islet arteriole contact. Histograms comparing islet size distribution (*x* axis) in islets with arteriole contact (blue) and without arteriole contact (red) across (**A**) human, (**B**) monkey, (**C**) pig, (**D**) rabbit, (**E**) ferret, and (**F**) mouse species. Insets: Density plots provide a representation of the distribution of islet size between 2 groups of islets with and without arteriole contact. The density plot function used in R/Python produces a visual, relative distribution of data for a given sample size, which allows for improved observation of data with differing sample sizes and distributions.

Islets with and without arteriole contact were examined for differences in size and number (or fraction size). On visualization of pancreatic sections, we found that islets with arteriole contact were visibly larger than those without arteriole contact, as shown in a representative image of human pancreatic tissue in Figure 5 (α-SMA–labeled arterioles are shown in yellow, islets with arteriole contact in blue, and islets without arteriole contact in red). In humans, islets with arteriole contact had a mean effective diameter of 104 ± 3 μm compared with 53 ± 1 μm in islets without arteriole contact (*P* < 0.05, Mann-Whitney *U* test). Similar data were obtained in the other 5 species examined, in which islets with arteriole contact were found to be significantly larger than those without arteriole contact at a significance level of *P* < 0.05. The proportions of islets with and without arteriole contact were as follows (percentage with/percentage without): human 21:79; monkey 40:60; pig 45:55; rabbit 18:82; ferret 14:86; and mouse 46:54. Data regarding islet mean diameter, size variability, and sample size are provided in Table 1. Interestingly, across all species, islets with arteriole contact had a larger variability in size compared with those without arteriole contact, as observed in the wider 95% CIs and larger SEM values and as demonstrated in the density plots comparing the distribution of both (Figure 6). The histograms in Figure 6 provide absolute numbers of islets in each effective diameter size range (*x* axis) observed in this study, whereas the density plots (inset) demonstrate a relative, visual representation of the distribution of islet size with respect to present (blue) or absent (orange) arteriole contact (Supplemental Figure 5). All species exhibited a right-sided tail in the distribution of islet size in islets with arteriole contact, demonstrating a size distribution weighted to include more larger-diameter islets, whereas islets without arteriole contact tended to be smaller, with less variability and tending toward a single mean diameter.

### Arteriole-capillary branching in the pancreas.

**Figure 7:**
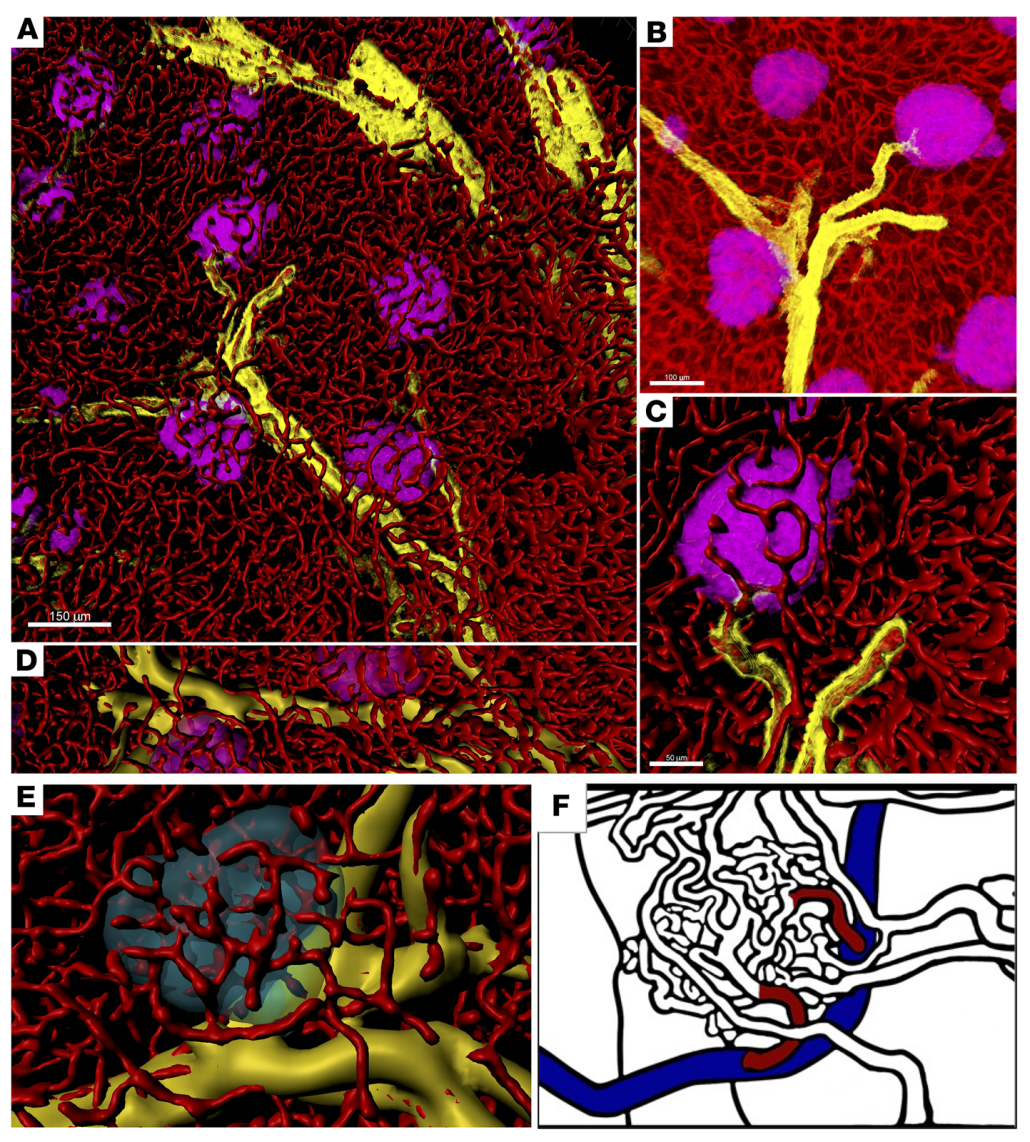
Arteriole-capillary branching in the pancreas. (**A**) Distribution of α-SMA. Pan-endocrine (magenta), α-SMA (yellow), and CD31 (red). Note that CD31-expressing endothelial cells are 3D surface rendered. Scale bar: 150 μm. (**B**) Enlarged view of the fluorescence image from **A**. Scale bar: 100 μm (**A** and **D**). (**C**) Enlarged view of a part of the image in **A**. Scale bar: 50 μm. (**D**) α-SMA–expressing arterioles are 3D surface rendered. (**E**) Representative islet contacting an arteriole: pan-endocrine (cyan), α-SMA (yellow), and CD31 (red). Note that the original image is shown in Figure 8E. (**F**) Graphical representation of the prevailing model of capillary branching from the arteriole (1, 27, 28). Blood vessels marked in red that are branched off from an arteriole (in blue) were denoted as “small arterioles” or “afferent vessels.”

The capillary networks in pancreatic islets have often been referred to as glomeruli (2) or glomerulus-like (5) due to their resemblance in size and a tuft of capillaries lined by fenestrated endothelia (32). However, the glomerulus is specialized for filtering blood, which enters and exits it through the afferent and efferent arterioles, respectively, under high pressure created by the difference in diameter between the proximal and distal ends of the glomerulus. These arterioles were elegantly visualized in 3D with the larger afferent arteriole and the smaller efferent arteriole by scanning electron microscopy of resin vascular casts (33). This group subsequently applied the same technique to visualize the pancreas, designating a few slightly larger capillaries around an islet as afferent vessels, while many other of the smaller ones were marked as efferent vessels (34, 35). Later, these afferent vessels were termed “small arterioles” (2). Arterioles branch from muscular arteries and consist of endothelium surrounded by a few layers of smooth muscle. α-SMA is normally restricted to cells of vascular smooth muscle. It is also expressed in large blood vessels (i.e., arteries and veins); however, the arteriole is defined as having a diameter of less than 100 μm (36). To further clarify the α-SMA expression here, pericytes strongly express NG2, PDGFRb, CD13, and CD146, but only weakly express α-SMA, if at all (37). In addition, pericytes reside within microvessels, whereas smooth muscle cells contribute to the vascular wall of larger vessels. In Figure 7A, the distribution of α-SMA is shown in yellow together with islets (magenta) and endothelial cells (red), with the latter being 3D surface rendered (Figure 7A). Smooth muscle cells surrounding arterioles are highlighted in distinction to capillaries, which are difficult to see in the original fluorescence image (Figure 7B). An enlarged view of a single islet is shown in Figure 7C. Once α-SMA was 3D surface rendered, it showed that capillaries were directly branched out from the arterioles (Figure 7D). The recent commercial availability of monoclonal antibodies against α-SMA (e.g., clone 1A4) (38) further enabled us to more effectively distinguish the arterioles shown in Figure 7E, in contrast to the prevailing model of vessel branching discussed above (Figure 7F) (1, 34, 35).

### Branched arterioles form regional units.

**Figure 8:**
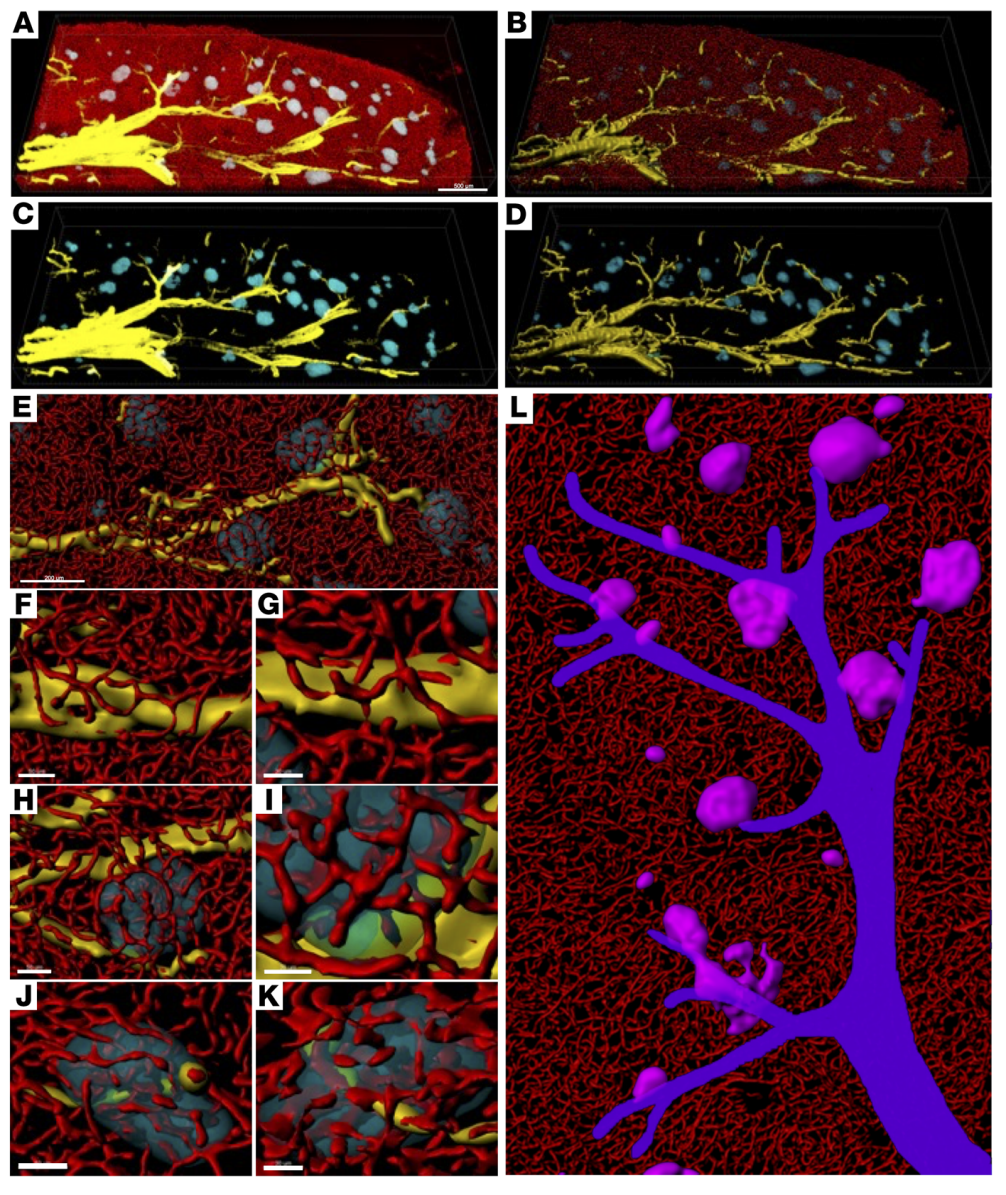
Branched arterioles form regional units. (**A**) Large area of a human pancreas. Fluorescence image: pan-endocrine cell marker (HPi1; cyan), α-SMA (yellow), and CD31 (red). (**B**) Surface-rendered image. (**C**) Fluorescence image without CD31 staining. (**D**) Surface-rendered image. Scale bars: 500 μm (**A**–**D**). (**E**) Close views of capillary branching directly from the arteriole. Scale bar: 200 μm. (**F**) A “unit” of branched arterioles. (**F** and **G**) Close views of arterioles. (**H** and **I**) Islet touching arterioles. (**J** and **K**) Arterioles passing through an islet. Scale bars: 50 μm (**F**, **H**, and **I**) and 30 μm (**G**, **J**, and **K**). (**L**) Schematic illustration showing regional blood supply through tree-like branched arterioles.

In summary, a large area of human pancreatic tissue from a 24-year-old male is shown, as an example, in Figure 8A, with islets stained with a pan–endocrine cell marker (HPi1) in cyan, capillaries (CD31) in red, and arterioles indicated by α-SMA in yellow (Figure 8, A–D). The 3D surface rendering shows a clear view of arterioles branching from a large vessel (Figure 8, B and D). A closer view of 1 arteriole branch shows islets clustering around it (Figure 8E). Capillaries directly branched from arterioles in exocrine tissues (Figure 8, F and G), to islets (Figure 8, H and I) and within islets (Figure 8, J and K), which suggests that this entire region can quickly detect changes in blood nutrient levels. This regional blood flow through tree-like arteriole branches is illustrated in Figure 8L (see also Supplemental Video 3).

## Discussion

The prevailing models of islet microcirculation are largely based on the common notion of the unique architecture of rodent islets that are believed to have a complete mantle of non–β cells surrounding the core of β cells. (2–6, 32). Most mouse islets are less than 150 μm in diameter (39). In humans, the majority of islets are 100–200 μm in diameter (30). Theoretically, to form a complete mantle around a core with a diameter of 100 μm, for example, the proportion of non–β cells required is over 50% (17). The proportions of β cells in mouse islets have been reported to be 60%–85% of islet endocrine cells (40–44), therefore rendering an intact mantle-core structure physically unlikely. The appearance of the mantle in 2D images might be explained by the principles of closure and continuity in Gestalt theory, which can influence human perception of complex situations such as the arrangement of α cells when interpreting results by filling in the gaps between cells (17).

Since the islet was long thought to be an enclosed structure that was organized into distinct regions with the β cell core and α and δ cells in the periphery, the direction of perfusion was speculated to be important in the regulation of hormone secretion (32). The Samols and Stagner group performed a series of anterograde (arterial) and retrograde (reversed or venous) infusion of antibodies directed against insulin, glucagon, or somatostatin ex vivo in various animal pancreata including human (45, 46). They concluded that insulin regulates glucagon secretion, which then regulates somatostatin secretion, suggesting a unidirectional regulation: β to α to δ cells. Somatostatin was regarded as “vascularly neutral,” owing to its downstream position in the sequence of cellular perfusion. These perfusion studies have been considered to reflect the same order of islet blood flow, which was based on rodent islet architecture (model 2) (32). However, in a similar experimental setting, Kawai et al. showed a comparable α, β, and δ cell response from arterial (via the celiac artery) and venous (via the portal vein) perfusion (47). The Huising group demonstrated interaction among all islet cell types, rather than specific unidirectional regulation (48, 49). They particularly clarified a pivotal role of δ cells with a number of their activators (e.g., glucagon, ghrelin, Glp-1, Ucn3, dopamine, leptin) and inhibitors (e.g., somatostatin, [nor]epinephrine, acetylcholine, palmitate/free fatty acids) (50). Gap junctions between β cells electrically connect endocrine interactions, and δ cells may influence these β cell gap junctions by altering electrical activity between cells or via electrical coupling (51–53).

Currently, the premise is that all the interactions mentioned above occur within the islet. We have recently shown that (a) the islet is not an enclosed structure; (b) pancreatic vasculature is integrated in its entirety; (c) Islet architecture has no relation to islet microcirculation; and (d) pancreatic microcirculation is bidirectional between the endocrine and exocrine pancreas (29). The present study further links endocrine and exocrine parts of the pancreas as a single organ through the integrated vascular network, where the arteriole feeds the whole pancreas regionally, as opposed to targeting individual islets. Direct capillary branching from the arteriole has revealed another unique feature of the pancreas that contrasts the general definition of the arteriole as being “a small branch of an artery leading into capillaries at its terminal ending” (27, 54). The new model of pancreas blood flow should provide insights for the integrated pathophysiology of the endocrine and exocrine pancreas. There may potentially be coordinated regulation between 2 compartments that would help clarify causal mechanisms of various pancreatic diseases including T1D, T2D, CFRD, T3cD, pancreatitis, pancreatic intraepithelial neoplasia, and pancreatic cancer. To address the limitations of the current study and to further our understanding in the field, studies such as in vivo recordings of pancreatic blood flow in large animals will obviously be required. It has been reported that radiologists are vigorously improving the imaging tools used for in vivo clinical imaging of the pancreas to identify possible ischemic insults in pancreatitis (55). Such technical advancements would shed light on the dynamic pancreas blood circulation.

## Methods

### Human pancreas specimens.

Human pancreata from donors were provided by the Gift of Hope Organ Procurement Organization (Chicago, Illinois, USA).

### Animal pancreas specimens.

The following animal pancreata specimens were provided by the Carlson Veterinary Clinic of the Animal Resource Center: monkey (rhesus macaques, 6.9–7.9 years old, both sexes); pig (Landrace-Yorkshire cross, 3.3 months old, female); rabbit (New Zealand white, 1.4 years old, female); ferret (8.6–10.8 months old, both sexes); and mouse (CD-1, 10 weeks old, both sexes).

### Antibodies.

The following primary antibodies were used: mouse monoclonal anti–pan-endocrine (Research Resource Identifier [RRID]: AB_1625452, HPi1; Novus Biologicals); mouse monoclonal anti-insulin (AB_2811080; Novus Biologicals); mouse monoclonal anti–human glucagon (AB_259852; MilliporeSigma); and mouse monoclonal anti–human CD31 (AB_314328, Biolegend). The primary antibodies were conjugated with a combination of amine-reactive fluorophores (*N*-hydroxysuccinimide-esters [NHS-esters]) (Thermo Fisher Scientific). Dylight 594–labeled tomato lectin from *Lycopersicon esculentum* (AB_2336416, Vector Laboratories); and mouse monoclonal anti–actin α-smooth muscle–Cy3 conjugate (AB_476856, MilliporeSigma) were used.

### 3D pancreas imaging.

The detailed method of 3D pancreas imaging was previously described (24). Briefly, a frozen pancreas tissue block (~5 mm in thickness) was fixed in 4% paraformaldehyde, embedded in 2% agarose gel, and mounted onto a vibratome. Sections (600–800 μm in thickness) were collected in cold PBS. These macrosections were then immunohistochemically stained overnight. Optical clearing was carried out by sequential incubation with 20%, 50%, 80%, and 100% (w/v) solutions of d-fructose and 0.3% (v/v) α-thioglycerol (MilliporeSigma) for 2 hours each and overnight in the last solution at 34°C with gentle agitation. Leica SP8 and Stellaris 8 laser scanning confocal microscopes (Leica Microsystems) were used to image tissue slices mounted between coverslips. 3D reconstruction and analysis were carried out using Fiji and Imaris software (Bitplane).

### Statistics.

Islet effective diameters were compared within species on basis of the presence or absence of arteriole contact. Differences in groups were compared using the Mann-Whitney *U* test, since data were not normally distributed based on results of the Shapiro-Wilk test for normality (*P* < 0.05 for all groups). Islet effective diameter was determined to be significantly different between groups if the Mann-Whitney *U* test resulted in a *P* value of less than 0.05. Density plots (Figure 6 insets) were calculated using the density function in R/Python, which uses a kernel density estimation to plot the density of features in a neighborhood around those features. Box plots (Supplemental Figure 5) were generated using the ggplot2 package in R. As described in the figure legend, the midline of the box plot is the median value, and the upper and lower edges of the box span the 25th–75th percentiles of the data. The distal ends of the whiskers represent 1.5 × the IQR, where the IQR is the 75th percentile value minus the 25th percentile value. The middle notches of the box plot roughly represent a 95% CI of the median as calculated by the median ± 1.57 × IQR/(*n*^0.5^), where *n* is the sample size (56).

### Study approval.

The use of deidentified human tissues in the study was approved by the IRB of the University of Chicago. All procedures involving animals were approved by the IACUC of the University of Chicago. For the use of human pancreas samples in this study, written informed consent was obtained from the donor or their next of kin. The specimens were all deidentified.

### Data availability.

Values for all data points in the graphs and plots are reported in the Supporting Data Values file.

## Author contributions

MH conceived the idea and designed the study. AAR, MPD, KCR, and MH performed experiments, analyzed data, and wrote the manuscript. MH, KCR, MPD, and MSR revised the manuscript. MH is the guarantor of this work and, as such, had full access to all the data in the study and takes responsibility for the integrity of the data and the accuracy of the data analysis.

## Supplementary Material

Supplemental data

Supplemental video 1

Supplemental video 2

Supplemental video 3

Supporting data values

## Figures and Tables

**Table 1:**
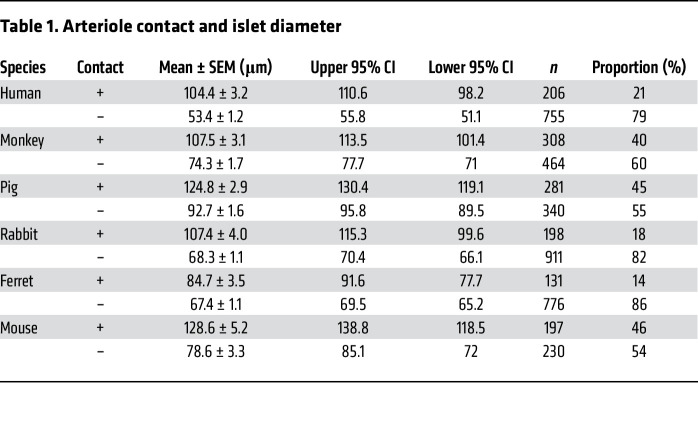
Arteriole contact and islet diameter

## Version 1 06/20/2023

In-Press Preview

## Version 2 08/01/2023

Electronic publication
