# Supplementary material for: Pancreatic regional blood flow links the endocrine and exocrine diseases
Source: J Clin Invest. 2023 Aug 1;133(15):e166185. doi: 10.1172/JCI166185 (PMC10378168; doi:10.1172/JCI166185)
Supplement: Supplemental data [file jci-133-166185-s050.pdf]

SUPPLEMENTARY FIGURES

Supplementary Figure S1. Large-scale 3D image analysis of the monkey pancreas.

Supplementary Figure S2. Large-scale 3D image analysis of the pig pancreas.

Supplementary Figure S3. Large-scale 3D image analysis of the rabbit pancreas.

Supplementary Figure S4. Large-scale 3D image analysis of the ferret pancreas.

Supplementary Figure S5. Boxplots visually representing islet size distribution in islets.

Supplementary Video 1. An example of an islet (cyan) that appears to have a dedicated arteriole (yellow) seen from one angle.

Supplementary Video 2. An example of three islets that none of them have a contact with the arteriole.

Supplementary Video 3. A close view of branching arterioles and islets shown in Fig.7.

Supplementary Figure S1

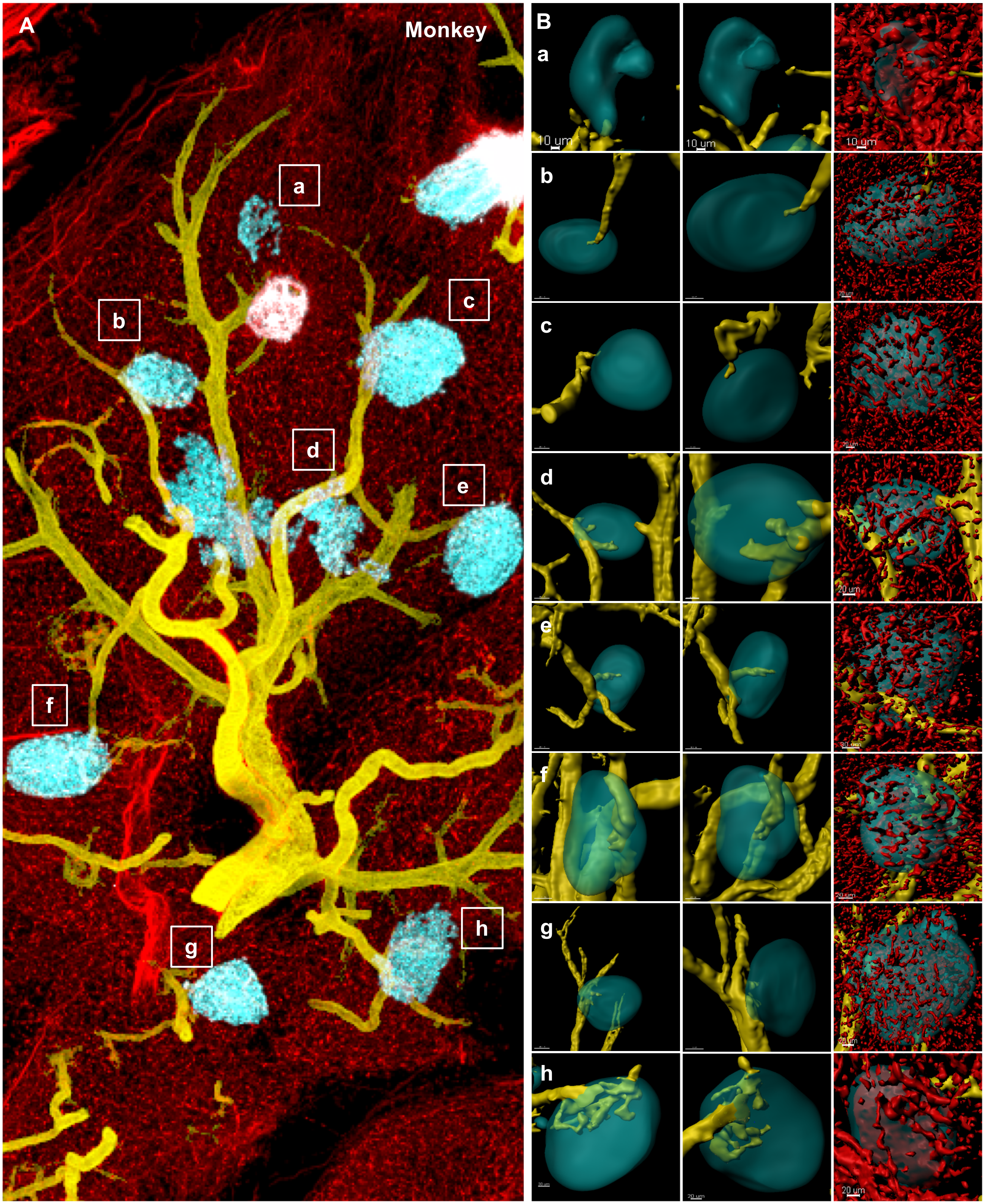

Supplementary Figure S1. Large-scale 3D image analysis of the monkey pancreas. A. Large-scale pancreatic tissue image showing pan-endocrine cell marker (HPi1; cyan), α- SMA (yellow) and CD31 (red). Scale bar: 200 μm. **B. a-h.** Individual islets corresponding to the labels in A. Islets, arterioles and capillaries are surface rendered. Scale bar (μm): a = all 10; b = 200, 30, 20; c = 200, 50, 20; d = 100, 200, 20; e = 30, 20, 20; f = 200, 50, 20; g = 200, 30, 20; h = 200, 100, 30.

Supplementary Figure S2

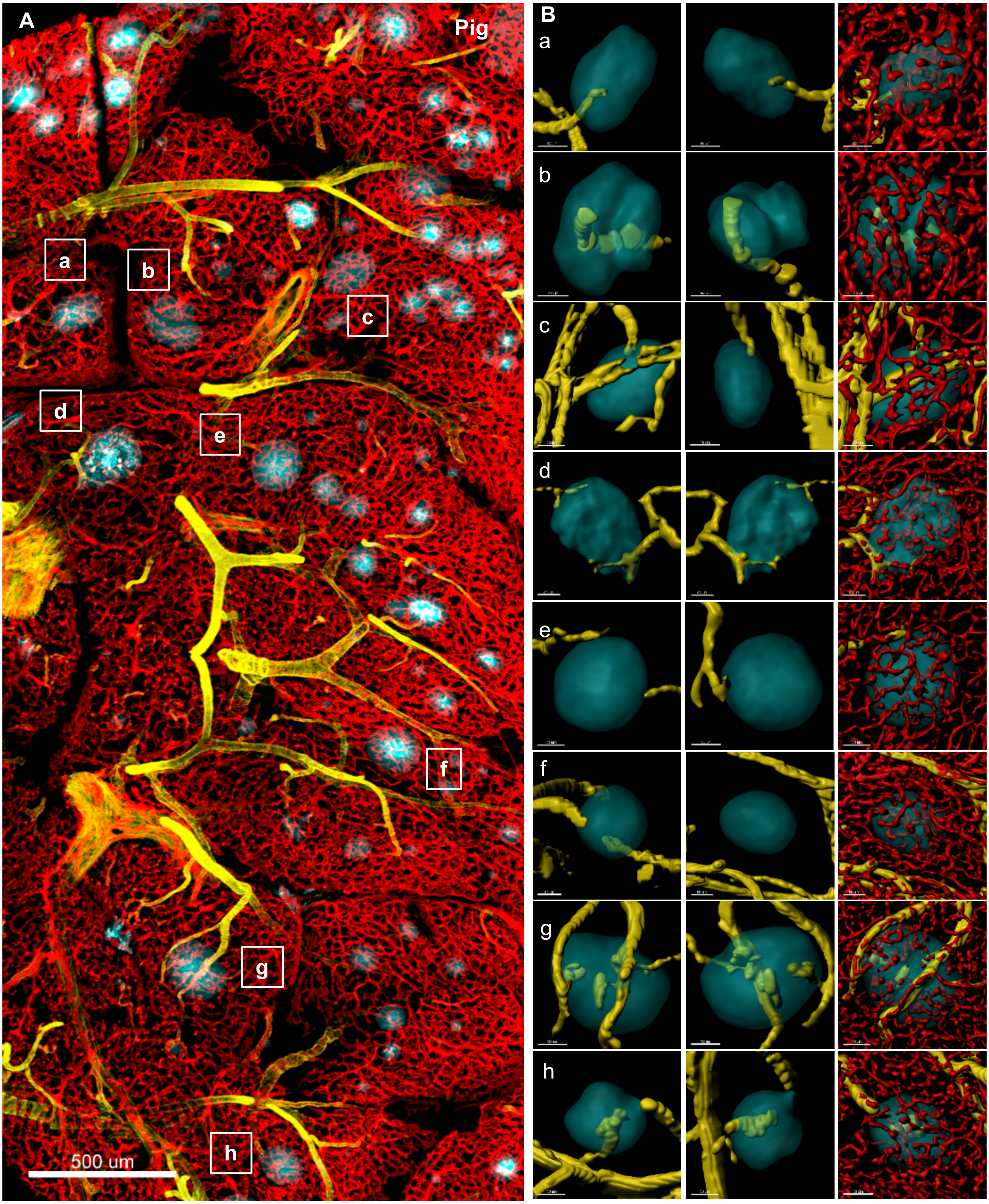

Supplementary Figure S2. Large-scale 3D image analysis of the pig pancreas. A. Large-scale pancreatic tissue image showing insulin, glucagon and somatostatin (cyan), α- SMA (yellow) and CD31 (red). Scale bar: 500 μm. **B. a-h.** Individual islets corresponding to the labels in A. Islets, arterioles and capillaries are surface rendered. Scale bar: all 50 μm.

Supplementary Figure S3

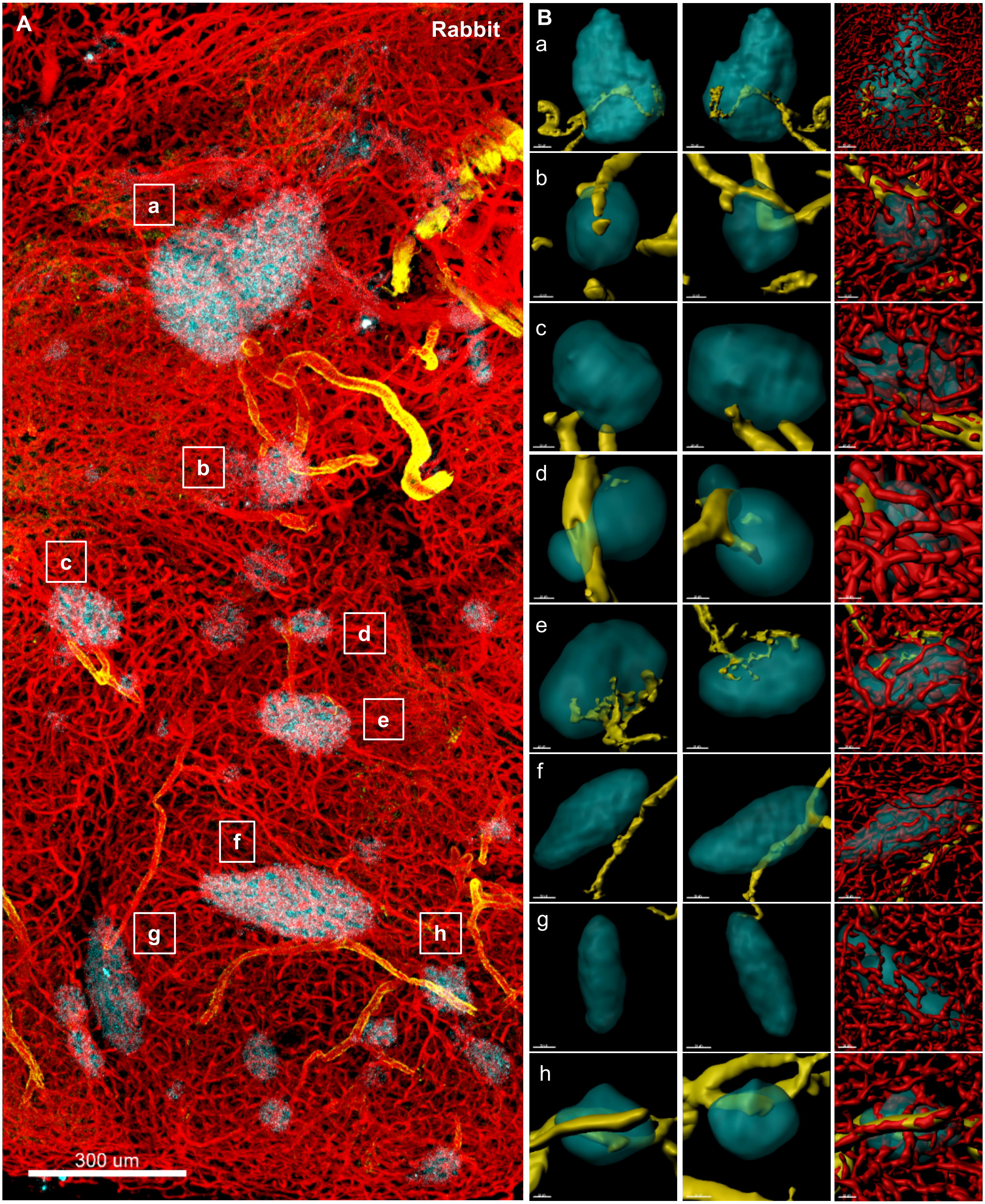

Supplementary Figure S3. Large-scale 3D image analysis of the rabbit pancreas. A. Large-scale pancreatic tissue image showing insulin, glucagon and somatostatin (cyan), α- SMA (yellow) and CD31 (red). Scale bar: 300 μm. **B. a-h.** Individual islets corresponding to the labels in A. Islets, arterioles and capillaries are surface rendered. Scale bar: a and f = 50 μm; b = 30 μm; c = 30 μm, 20 μm, 20 μm; d = 20 μm; e = 20 μm, 30 μm, 30 μm; g = 50 μm, 50 μm, 30 μm; h = 20 μm, 30 μm, 20 μm.

Supplementary Figure S4

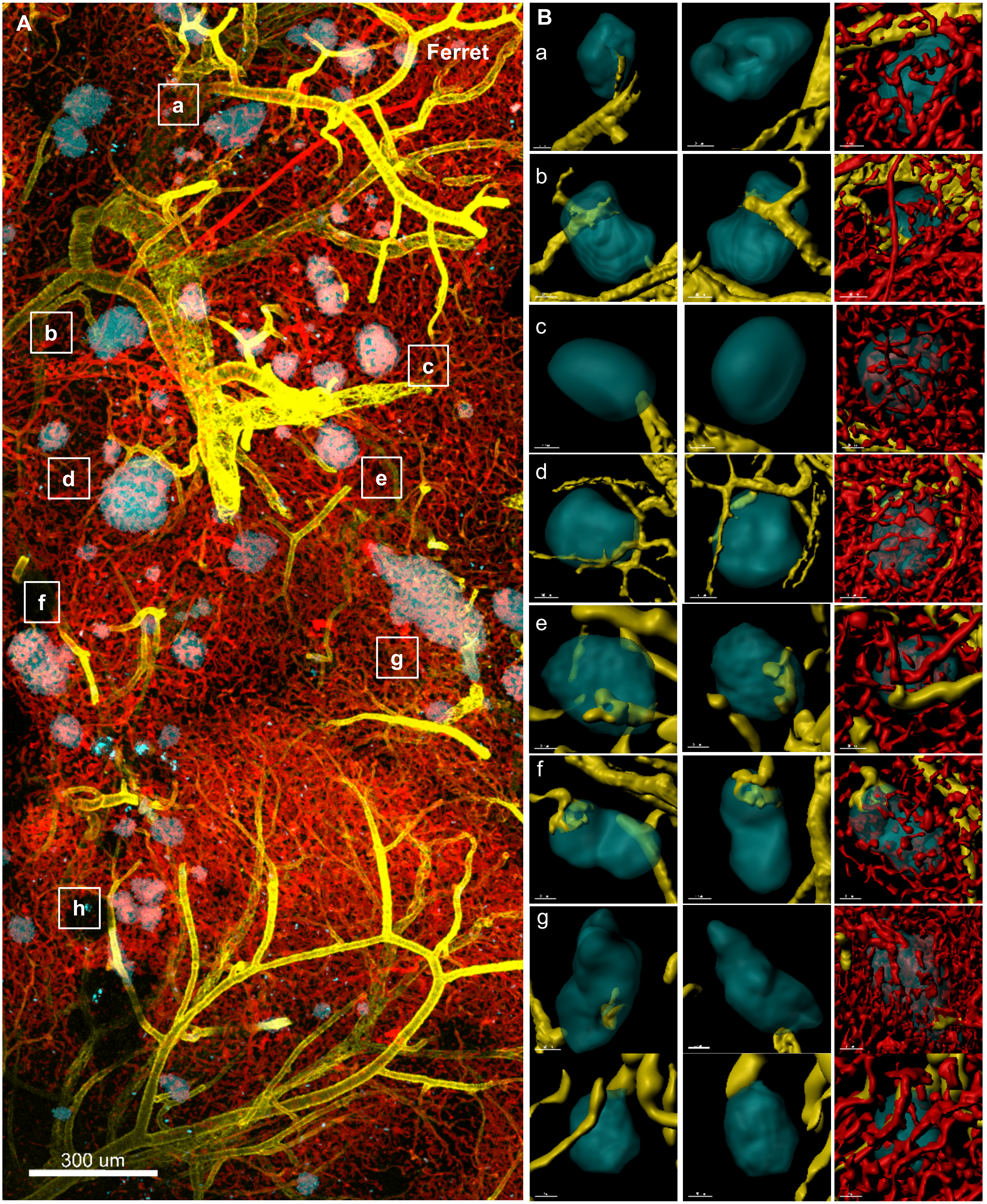

Ferret

Supplementary Figure S4. Large-scale 3D image analysis of the ferret pancreas. A. Large-scale pancreatic tissue image showing insulin, glucagon and somatostatin (cyan), α-SMA (yellow) and CD31 (red). Scale bar: 300 μm. **B. a-h.** Individual islets corresponding to the labels in A. Islets, arterioles and capillaries are surface rendered. Scale bar: a, c, and f = 30 μm; b = 30 μm, 30 μm, 50 μm; d and g = 50 μm; e = 20 μm, 20 μm, 30 μm; h = 20 μm.

Supplementary Figure S5

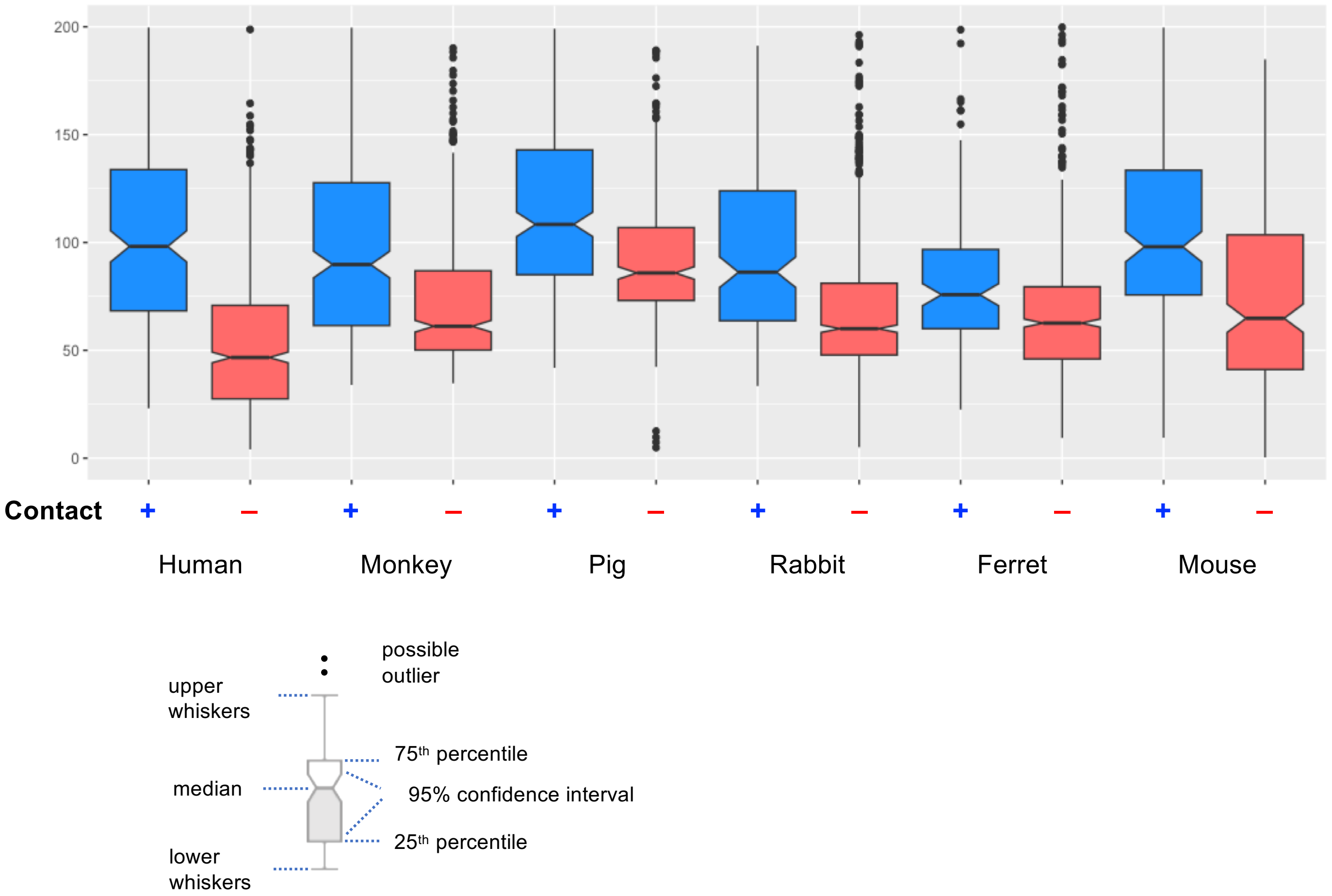

Supplementary Figure S5. Boxplots representing islet size distribution in islets. Islets with arteriole contact (blue) and islets without arteriole contact (red) in human, monkey, pig, rabbit, ferret, and mouse. The y-axis measures effective islet diameter in µm. The midline of the boxplot represents the median value. The 75th and 25th percentile values are represented by the top and bottom edges of the box, respectively. The upper edge of the boxplot whisker is represented by the 75th percentile value + 1.5*IQR, where IQR is the inter-quartile range (75th percentile value – 25th percentile value). The lower edge of the boxplot whisker is represented by the 25th percentile value – 1.5*IQR. Outlier values beyond the upper and lower bounds of the whiskers are represented by black dots. The middle notch of the boxplot roughly represents a 95% confidence interval of the median, with range median +/- 1.57*IQR/($n^{0.5}$), where n is sample size. There are 114 outlier data points not represented in the plot as they extend beyond the bounds of the y-axis.
